# *KRAS* Assessment Following ESMO Recommendations for Colorectal Liver Metastases. Is It Always Worth It?

**DOI:** 10.3390/healthcare10030472

**Published:** 2022-03-03

**Authors:** Olga Morató, Maria Villamonte, Patricia Sánchez-Velázquez, Eva Pueyo-Périz, Luís Grande, Benedetto Ielpo, Edoardo Rosso, Alessandro Anselmo, Fernando Burdío

**Affiliations:** 1 Department of Surgery, Division of Hepato-Biliary and Pancreatic Surgery, Hospital del Mar, Medical Research Institute (IMIM), University Pompeu Fabra, 08003 Barcelona, Spain; maryvr31@hotmail.com (M.V.); patri_sv5@hotmail.com (P.S.-V.); epueyoperiz@gmail.com (E.P.-P.); LGrande@parcdesalutmar.cat (L.G.); Ielpo.b@gmail.com (B.I.); fburdio@hotmail.com (F.B.); 2 Unité des Maladies de l’Appareil Digestif et Endocrine, Department of Surgery and Robotics, Centre Hospitalier de Luxembourg, L-1210 Luxembourg, Luxembourg; edoardo_rosso@hotmail.com; 3 HPB and Transplant Unit, Department of Surgery, Policlinico Tor Vergata, 00173 Rome, Italy; alessandroanselmo.ptv@gmail.com

**Keywords:** *KRAS* oncogene, colorectal liver metastases, tumour burden score, synchronic tumours

## Abstract

Background: Genetic evaluation is essential in assessing colorectal cancer (CRC) and colorectal liver metastasis (CRLM). The aim of this study was to determine the pragmatic value of *KRAS* on oncological outcomes after CRLM according to the ESMO recommendations and to query whether it is necessary to request *KRAS* testing in each situation. Methods: A retrospective cohort of 126 patients who underwent surgery for hepatic resection for CRLM between 2009 and 2020 were reviewed. The patients were divided into three categories: wild-type *KRAS*, mutated *KRAS* and impractical *KRAS* according to their oncological variables. The impractical (not tested) *KRAS* group included patients with metachronous tumours and negative lymph nodes harvested. Disease-free survival (DFS), overall survival (OS) and hepatic recurrence-free survival (HRFS) were calculated by the Kaplan–Meier method, and a multivariable analysis was conducted using the Cox proportional hazards regression model. Results: Of the 108 patients identified, 35 cases had *KRAS* wild-type, 50 cases had a *KRAS* mutation and the remaining 23 were classified as impractical *KRAS*. Significantly longer medians for OS, HRFS and DFS were found in the impractical *KRAS* group. In the multivariable analyses, the *KRAS* mutational gene was the only variable that was maintained through OS, HRFS and DFS. For HRFS (HR: 13.63; 95% confidence interval (CI): 1.35–100.62; *p* = 0.010 for *KRAS*), for DFS (HR: 10.06; 95% CI: 2.40–42.17; *p* = 0.002 for *KRAS*) and for OS (HR: 4.55%; 95% CI: 1.37–15.10; *p* = 0.013). Conclusion: Our study considers the possibility of unnecessary *KRAS* testing in patients with metachronous tumours and negative lymph nodes harvested. Combining the genetic mutational profile (i.e., *KRAS* in specific cases) with tumour characteristics helps patient selection and achieves the best prognosis after CRLM resection.

## 1. Introduction

Colorectal cancer (CRC) is the third most common cancer worldwide in terms of incidence (6.1%), and second worldwide in terms of mortality (9.2%) [1,2]. It is estimated that 15–25% of CRC patients will have developed metastases at the time of primary diagnosis (synchronic tumours), associated with poor prognosis [3,4], while another 25% of patients will develop metastases in 5 years, half of these will settle into the liver [5,6].

Fast forwarding to today, there has been a remarkable enhancement in overall survival (OS) for colorectal liver metastases (CRLM). Effective targeted chemotherapies, biological agents combined with technically advanced resection plans, have had proven survival benefits [7,8,9,10,11,12]. Apart from this, tumour morphology and colorectal primary features are recognized as independent OS predictors after CRLM resection [6,13,14]. The well-known clinicopathological scores by Fong et al. [15] (clinical risk score) and Nordlinger et al. [16] are widely used as OS predictors, while the tumour burden score (TBS) arose as a new robust predictor of long-term survival [17,18].

Novel, more complex biomarkers of tumour biology are also emerging, which, in combination with tumour morphological features, facilitate the prospective assessment of outcomes and follow-up response to treatment after CRLM resection [18,19]. Some studies claim that site-specific patterns of CRC metastases have an impact on patient’s outcomes [5] and *KRAS* mutations play a (key) role in these patterns [20,21,22]. *KRAS* mutations affect the risk of recurrence and survival in patients who undergo CRLM resections [19,21,23]. They are known to have more aggressive tumour biology than wild-type [22,24,25] and have also been associated with a lower likelihood of having resectable CRLM [26,27]. *KRAS* and BRAF, the most widely studied oncogene mutations for CRC, should be an indispensable part of tumour analysis [8]. *KRAS* mutated affects recurrence risk and survival in patients who undergo complete liver resection for CRLM [19,20,21] by involving them in the surveillance [22].

Although ESMO guidelines for CRLM management recommendations have changed over the last 10 years as the understanding of genetics has developed [28,29,30], ESMO’s and other national societies’ [31] recommendations support patients with a CRC metastatic disease *KRAS* assessment. Nevertheless, it remains controversial whether *KRAS* testing of CRC is better practiced as a “reflex” or an “on demand” process [26]. The “on demand” process aims to group high-risk features on resected CRCs (those with extramural vascular invasion, nodal metastases and/or a pT4 stage), which is linked to the different biological characteristics synchronous and metachronous liver metastases appear to contain [32].

As such, the objective of the current study was to determine whether *KRAS* mutational status according to ESMO’s CRLM patient-management recommendations in all cases provides a better OS rate, DFS and hepatic recurrence. We sought to better understand oncogene and tumour characteristics allowing us to determine the long-term patient prognosis more accurately.

## 2. Methods

### 2.1. Study Design

**Figure 1:**
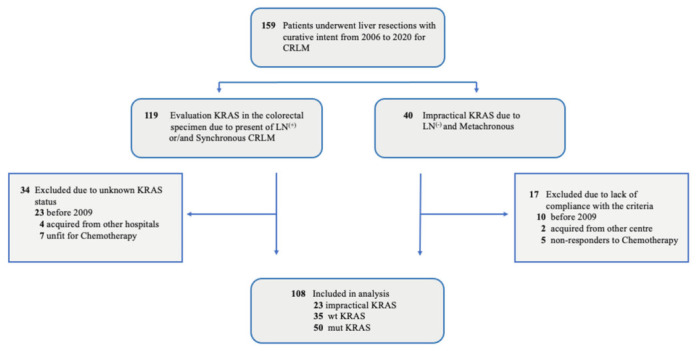
Study Flowchart: CRLM indicates colorectal liver metastases; LN, lymph nodes positive (+) or negative (−); mut *KRAS,* mutated genotype; wt *KRAS*, wild-type genotype.

From September 2009 to February 2020, all the consecutive patients who underwent curative-intent surgery for CRLM at the HPB unit in the Hospital del Mar’s IMIM (Medical Research Institute) were included. Only those who underwent ablation, or a palliative liver resection (R2 resection) were excluded. Figure 1 contains a flowchart of all those included in the retrospective study. The 126 patients were divided into two groups according to our centre’s oncological protocol, which started in 2009. The protocol tested *KRAS* “on demand” only in patients with resected CRCs and high-risk features such as those with a synchronous tumour (<6 months) and/or positive lymph nodes harvested. The impractical (not tested) *KRAS* group included patients with metachronous tumours (>6 months after CRC diagnoses) and negative lymph nodes harvested. In this group *KRAS* was not evaluated as it was considered to be unnecessary [33]. According to our protocol, *KRAS* was only evaluated in 96 patients and the other 30 patients, classified as impractical *KRAS*, were not evaluated. Based on the ESMO evidence [28] 10 years ago, genomic DNA was isolated from either primary tumour or CRLM tissue specimens and was used as a template for sequencing *KRAS* codons [30]. Eighteen of these 126 patients were excluded.

Subsequently, 108 patients were finally included in the analysis and divided into 3 categories according to their oncologic biomarker status: *KRAS* wild-type (absence of the mutation), *KRAS* mutated and impractical (not tested) *KRAS*. Of these, 50 patients had *KRAS* mutated, 35 had *KRAS* wild-type and the remaining 23 were impractical *KRAS* (not tested).

The study was conducted in accordance with the principles of the Declaration of Helsinki, the Good Clinical Practice Guidelines and was approved by the hospital’s Clinical Research and Ethics Committee approved. All the patients gave their written informed consent prior to surgery and patient data were collected through a prospectively maintained institutional database. This study followed the Strengthening the Reporting of Observational Studies in Epidemiology, Guidelines STROBE.

### 2.2. Parameters Studied

We collected standard demographic, clinicopathologic and genetic variables, including age, sex and characteristics of the extension of the primary CRC including the American Joint Committee on Cancer (AJCC) (T) stage, and the presence or absence of lymph node metastasis (*n*). The stages were evaluated in each category according to the TNM latest AJCC (8th edition). The characteristics of the resected liver specimen were diameter of largest tumour, number of hepatic tumours using the final histopathological report (including microsatellite lesions) and the TBS. We also collected data on the type of surgery conducted, whether anatomical or atypical, and margins of the pathological specimen, with R1 defined as microscopically positive resection margins [34]. Chemotherapy was evaluated in pre or postoperative terms, cetuximab whether it had been administered, and synchronous (<6 months) vs. metachronous (>6 months) presentation of liver disease.

The primary endpoints of this study were the assessment of OS, disease-free survival (DFS) and hepatic recurrence-free survival (HRFS) in terms of *KRAS* and TBS. OS was calculated for each patient from the date of surgery to the date of death or last follow-up. Similarly, DFS and HRFS were calculated from the date of resection until the first radiologic or pathologic evidence of recurrence or, in the case of no recurrence, until the date of the last follow-up.

### 2.3. Calculation of Tumour Burden Score

The secondary endpoint was found by evaluating the morphological tumour features through a survival score, TBS. TBS was defined as the distance from the origin on a Cartesian plane that incorporated 2 variables: maximum tumour size (x-axis) and number of liver lesions (y-axis). The Pythagorean theorem was used to calculate the distance of any given point from the origin of the plane, whereby (TBS^2^ = (maximum tumour diameter)^2^ + (number of liver lesions)^2^) [17,35].

### 2.4. Statistical Analysis

The Kolmogorov–Smirnov test was used to assess the normal distribution of data. Continuous variables were expressed as the median (interquartile range) and compared by the Kruskal–Wallis test. Categorical variables were expressed as absolute numbers and compared using the chi-square test. Survival curves such as HRFS, DFS and OS were estimated using the Kaplan–Meier method, with comparisons of three categories (*KRAS* mutated, *KRAS* wild-type and Impractical *KRAS*-not tested) by the log-rank test. A multivariate landmark analysis was conducted using the Cox proportional hazards regression model to identify independent prognosis predictors in multivariable analysis. Hazard ratios (HR) were reported with 95% confidence intervals (95% CI), as appropriate. Factors found to be significant predictors of univariate analysis were subjected to multivariate analysis using the Cox proportional hazards model (backward stepwise regression analysis).

Differences in variables were significant at a threshold of *p* < 0.05. The statistical analyses were carried out on SPSS version 25.0 (SPSS, Chicago, IL, USA).

## 3. Results

### 3.1. Patient Characteristics

Baseline characteristics of the 108 patients who underwent hepatic resection did not differ significantly between the patients with *KRAS* wild-type, *KRAS* mutated or impractical *KRAS* (not tested) in terms of age and sex. Regarding CRLM characteristics, impractical *KRAS* (not tested) was significantly more likely to be less aggressive in the early stages, such as Stages II and III, according to AJCC-UICC. Although the median number of metastatic lesions was similar, the median size of the largest metastatic lesions was significantly smaller in the wild-type *KRAS*, and TBS was significantly larger in the *KRAS* mutated group. There was no difference among groups according to R1 margin resections, although *KRAS* mutated had more patients with postoperative chemotherapy (*p* = 0.05) (Table 1).

### 3.2. Cumulative Incidence of Recurrence and Survival According to Different KRAS Expression

**Figure 2:**
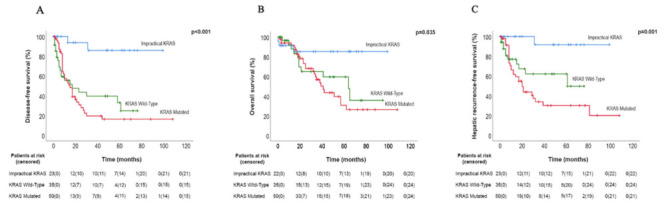
(**A**) The disease-free survival (DFS) rate is shown for patients with *KRAS* wild-type versus *KRAS* mutated versus Impractical *KRAS* tumours. (**B**) The overall survival (OS) rate is shown for patients with *KRAS* wild-type versus *KRAS* mutated versus Impractical *KRAS* tumours. (**C**) The hepatic recurrence rate is shown for patients with *KRAS* wild-type versus *KRAS* mutated versus Impractical *KRAS* tumours. Truncates at 120 months. 95% CI indicates 95% confidence interval.

The 5-years DFS rate for patients with the impractical *KRAS* (not tested), *KRAS* wild-type and *KRAS* mutated were 85.9%, 33.1% and 16.3% respectively (*p* < 0.0001). For HRFS, the 5-year rate was even more striking at that point, in which impractical *KRAS* (not tested), *KRAS* wild-type and *KRAS* mutated were 91.7% 62.1% and 30.1% respectively (*p* = 0.001). The 5-year OS rates for patients with impractical *KRAS* (not tested), *KRAS* wild-type and *KRAS* mutated were 85.2%, 59.5% and 30.7% respectively (*p* = 0.035) (Figure 2).

### 3.3. Uni-Multivariable Analysis among DFS, HRFS and OS

Some factors in the univariable analysis were associated with longer DFS (Table 2): age, Stages II or III, synchronous CRLM tumours, TBS, tumour number, *KRAS* status, R1 margin and preoperative chemotherapy. When we focus on HRFS, the same variables as in the DFS had links with a higher risk of HRFS, except for the largest tumour size, R1 margins and chemotherapy. In the univariable analysis, only three factors were independent predictors of OS, synchronous CRLM tumours, *KRAS* mutated and postoperative chemotherapy. However, only TBS and *KRAS* (mutated and wild-type) were maintained in the DFS multivariable analysis as independent factors, the same HRFS independent factors related to the multivariable analysis (Table 3). Nevertheless, when we looked at the multivariable analyses of OS, apart from *KRAS* mutated remaining as an independent factor, TBS no longer played a key role and was no longer an independent variable, which was instead postoperative chemotherapy (Table 4)**.** The *KRAS* mutational gene was the only variable that survived three times in the multivariable analysis.

## 4. Discussion

The main finding in our analysis appeared as we made further progress in accepting impractical *KRAS* (patients not tested) as a new entity to be considered as different from *KRAS* mutated or even *KRAS* wild-type patients. This interesting finding highlights the idea that testing *KRAS* as a “reflex” just creates a possibility of unnecessary testing of CRC tissue from patients who never developed metastases [26]. Surprisingly, after analysing and comparing the impractical *KRAS* (those with metachronous and negative lymph node tumours) with *KRAS* mutated or *KRAS* wild-type patients, the difference in terms of HRFS, DFS and OS sharply increased over the others. On the other hand, testing “on demand” means the patients who may benefit from new treatments such as anti-EGFR can be attended. According to the ESMO recommendation for patients with CRLM, anti-EGFR therapies should only be considered for patients with *KRAS* wild-type. However, it is noteworthy that the ESMO recommendations were being changed in accordance with new times and new genetic knowledge and research.

The exact biological course of the synchronous and metachronous liver metastases is still unknown; however, a review of the literature confirms that they exhibit different biological characteristics to their respective CRC primary tumours [36,37], such as the reduction in p27 expression in the metachronous group, suggesting that there is a “posttranslational” degradation of the proteins in the liver metastases [32], or Kim et al. [32,38] finding a higher expression of VEGF within the synchronous metastases group than in the CRC primary tumour. Others found different immunological response cells in the metachronous group, suggesting a need to clarify whether both groups had to be underlined at the same level. Indeed, it has been accepted that a better understanding of the biological behaviour of tumour biology is a more important factor in survival than surgical margin clearance in the era of modern chemotherapy regimens [39,40].

*KRAS* is one of the most commonly employed surrogates of genetic alteration in CRC and has been associated with an increased rate of vascular invasion and hematogenous metastasis [20]. *KRAS* is an oncogene located downstream of the epidermal growth factor receptor (EGFR), which is the target for anti-EGFR treatment such as cetuximab. Interestingly, detection of *KRAS* tumours’ mutational status is predictive as a negative marker and the patient is unlikely to benefit from EGFR antibody therapy (cetuximab, bevacizumab and panitumumab), so that patients with *KRAS* wild-type status seem to respond better to anti-EGFR treatment.

The PRIME study, a randomized control trial, supports a positive benefit–risk profile for panitumumab-FOLFOX4 in patients with previously untreated wild-type *KRAS* [41]. Nevertheless, another recent RCT between chemotherapy alone or the combination of cetuximab and chemotherapy found that cetuximab seemed to be detrimental to patients with *KRAS* wild-type in exon 2, with shorter progression-free survival [42]. *KRAS* genotyping to guide anti-EGFR therapy is evolving rapidly and is being updated. There are some reports of RAS wild-type CRC patients who first show a response to EGFR inhibitors and later demonstrate RAS mutations with progressive disease, suggesting an acquired resistance to these drugs [43]. These findings highlight the imperative need to go deeper into the different genetic evaluations, such as “liquid biopsies”, which could play a better role in detecting emerging RAS mutant clones in the near future [26,44]. Unfortunately, our study is retrospective and genotypes could not be determined.

The data strongly suggested that the course of CRLM in the patients depends on both tumour morphology, such as TBS, and the genetic mutational profile [18]. A tumour burden “metro ticket” score based on final pathology has recently been proposed to predict outcomes following CRLM resection [17]. The TBS and *KRAS* mutation category play a key role in the univariate analysis in providing an increased risk of recurrence. In the multivariable analysis, they are still independent regarding DFS and HRFS; however, they are no longer independently represented in OS. These findings support the fact that both TBS and *KRAS* are good predictors of survival outcomes after CRLM resection and should be under consideration before surgery is performed.

The current study had several limitations, which ought to be considered when deciphering these results. For instance, given its retrospective design, it could have suffered from selection bias. It should also be noted that *KRAS* mutational status was resolved either through analysis of the metastasis or of the primary tumour, depending on specimen accessibility. As such, it is possible that the mutational status differed between the two tumour sites.

It should also be noted that this analysis did not take into account potential differences among different *KRAS* mutation variants because they were not differentiated in the available literature [19]. In addition, other potentially important molecular biomarkers such as BRAF, PI3K and TP53 were not evaluated. Lastly, as the total numbers of patients and impractical *KRAS* patients included are small, this could introduce another bias factor into the analysis.

## 5. Conclusions

Despite two decades-worth of data, the debate over whether tumour biology should be given more consideration than tumour characteristics among patients with resectable CRLM has not ended. Requesting *KRAS* makes sense “as a demand” for those patients with more aggressive tumours and should be considered today as an indispensable part of decision making before embarking on aggressive surgical therapy, while in other patients with specific criteria, the *KRAS* testing could be omitted. Our study emphasizes the importance of considering the assessment of tumour characteristics by the TBS score within a *KRAS* evaluation in synchronous and aggressive CRC, looking towards a lower recurrence and longer survival.

## Figures and Tables

**Table 1:** Clinical Characteristics and Pathologic Features of *KRAS*-Mutant, *KRAS*-Wild-Type and Impractical *KRAS* in Colorectal Cancer Cases.

Characteristic	KRAS Wild-Type (*n* = 35)	KRAS Mutated (*n* = 50)	Impractical KRAS (Not Tested) (*n* = 23)	*p*-Value
Patient characteristics				
Age (year) ^a^	71 (63–76)	69 (58–73)	70 (66–76)	0.281 ^b^
Female sex	14 (40.0%)	16 (32.0%)	9 (39.1%)	0.709 ^c^
Primary tumour characteristics				
T3 or T4	30 (85.7%)	46 (92.0%)	18 (78.3%)	0.444 ^c^
N1–N2				
CRLM characteristics	26 (74.3%)	40 (80.0%)	0 (0%)	<0.0001 ^c^
Stage II or III	6 (17.1%)	17 (34.0%)	18 (78.3%)	<0.0001 ^c^
Synchronous CRLM	29 (82.9%)	33 (66.0%)	0 (0%)	<0.0001 ^c^
Tumour Burden Score ^a^	3.4 (2.1–4.9)	4.0 (2.9–6.2)	3.5 (2.7–5.2)	0.048 ^b^
Tumour number ^a^	2 (1.0–3.0)	2 (1.0–3.3)	1 (1.0–3.0)	0.159 ^b^
Size of largest tumour size (cm) ^a^	2 (1.5–3.6)	2.8 (2.0–5.1)	2.9 (2.2–4.2)	0.040 ^b^
Surgery procedure				
Anatomical	12 (34.3%)	28 (56.0%)	9 (39.1%)	0.112 ^c^
Atypical	23 (65.7%)	22 (44.0%)	14 (60.9%)	
R1 margin resection status	9 (25.7%)	19 (38.0%)	4 (17.4%)	0.166 ^c^
Chemotherapy				
Preoperatively	9 (25.7%)	10 (20.0%)	5 (21.7%)	0.822 ^c^
Postoperatively	21 (60.0%)	33 (66.0%)	6 (26.1%)	0.05 ^c^
Cetuximab preoperatively	0 (0%)	3 (6.0%)	0 (0%)	0.165 ^c^
Cetuximab postoperatively	1 (2.8%)	5 (10.0%)	0 (0%)	0.182 ^c^

CRLM, colorectal liver metastases. ^a^ Continuous variables are expressed as median (interquartile range). Categorical variables are expressed as absolute numbers and percentages. ^b^ Kruskal–Wallis test, ^c^ Chi-square test. Differences in variables were significant at a threshold of *p* < 0.05.

**Table 2:** Uni-and Multivariable Predictors of Disease-Free Survival in the Entire Cohort.

Prognostic Factor		Univariable Analysis		Multivariable Analysis	
		HR (95% CI)	*p*-Value	HR (95% CI)	*p*-Value
Patients’ characteristics					
	Age (year)	0.98 (0.95–0.99)	**0.039**	0.99 (0.96–1.02)	0.578
	Sex (Male/Female)	0.97 (0.56–1.66)	0.905		
Primary tumour characteristics					
	T1–T2/T3–T4	1.05 (0.26–4.33)	0.942		
	N0/N1–N2	2.26 (1.23–4.15)	**0.008**	0.93 (0.49–1.77)	0.829
	Stage II or III	2.69 (1.44–5.01)	**0.002**	1.21 (0.61–2.41)	0.581
CRLM characteristics					
	Synchronous CRLM	3.18 (1.71–5.93)	**<0.001**	1.54 (0.76–3.11)	0.235
	Tumour Burden Score	1.13 (1.06–1.21)	**<0.001**	1.12 (1.04–1.20)	**0.001**
	Tumour number	1.16 (1.08–1.25)	**<0.001**	1.06 (0.94–1.20)	0.370
	Size of largest tumour size (cm)	1.08 (0.98–1.18)	0.131		
*KRAS* status					
	Impractical *KRAS* (not tested)	1		1	
	*KRAS* Wild-Type	9.39 (2.19–40.24)	**0.003**	9.57 (2.23–41.04)	**0.002**
	*KRAS* Mutated	11.17 (2.67–46.70)	**0.001**	10.06 (2.40–42.17)	**0.002**
Surgery procedure					
	Atypical/Anatomical	1.16 (0.69–1.96)	0.568		
R1 margin resection status (yes/no)		1.94 (1.13–3.34)	**0.017**	1.52 (0.81–2.83)	0.189
Chemotherapy (yes/no)					
	Preoperatively	1.71 (1.01–2.89)	**0.044**	0.97 (0.56–1.68)	0.909
	Postoperatively	1.05 (0.60–1.86)	0.856		

CRLM, colorectal liver metastases; HR, Hazard ratio; CI, confidence interval. Bold values are statistically significant (*p*-value < 0.05).

**Table 3:** Uni-and Multivariable Predictors of Hepatic Recurrence in the Entire Cohort.

Prognostic Factor		Univariable Analysis		Multivariable Analysis	
		HR (95% CI)	*p*-Value	HR (95% CI)	*p*-Value
Patients’ characteristic					
	Age (year)	0.96 (0.94–0.99)	0.011		
	Sex (Male/Female)	1.01 (0.54–1.89)	0.985		
Primary tumour characteristics					
	T1–T2/T3–T4	0.63 (0.15–2.63)	0.528		
	N0/N1–N2	2.24 (1.10–4.60)	**0.027**	1.08 (0.48–2.44)	0.853
	Stage II or III	1.94 (0.97–3.89)	0.060		
CRLM characteristics					
	Synchronous CRLM	2.25 (1.12–4.49)	**0.022**	0.93 (0.42–2.04)	0.855
	Tumour Burden Score	1.18 (1.10–1.27)	**<0.0001**	1.16 (1.08–1.25)	**<0.0001**
	Tumour number	1.20 (1.10–1.30)	**<0.0001**	0.95 (0.64–1.41)	0.800
	Size of largest tumour size (cm)	1.12 (1.01–1.23)	**0.027**	0.87 (0.55–1.39)	0.565
*KRAS* status					
	Impractical *KRAS* (not tested)	1		1	
	*KRAS* Wild-Type	9.53 (1.23–73.91)	**0.031**	9.44 (1.21–73.34)	**0.032**
	*KRAS* Mutated	15.77 (2.14–116.17)	**0.007**	13.63 (1.35–100.62)	**0.010**
Surgery procedure					
	Atypical/ Anatomical	1.01(0.55–1.87)	0.973		
R1 margin resection status (yes/no)		1.67 (0.88–3.17)	0.117		
Chemotherapy (yes/no)					
	Preoperatively	1.60 (0.86–2.98)	0.137		
	Postoperatively	1.56 (0.75–3.28)	0.238		

CRLM, colorectal liver metastases; HR, Hazard ratio; CI, confidence interval. Bold values are statistically significant (*p*-value < 0.05).

**Table 4:** Uni-and Multivariable Predictors of Overall Survival in the Entire Cohort.

Prognostic Factor		Univariable Analysis		Multivariable Analysis	
		HR (95% CI)	*p*-Value	HR (95% CI)	*p*-Value
Patients’ characteristic					
	Age (year)	1.00 (0.97–1.04)	0.805		
	Sex (Male/Female)	0.49 (0.24–0.99)	**0.049**	1.96 (0.95–4.04)	0.680
Primary tumour characteristics					
	T1–T2/T3–T4	0.52 (0.12–2.18)	0.370		
	N0/N1–N2	1.34 (0.70–2.57)	0.386		
	Stage II or III	2.45 (1.17–5.16)	**0.018**	0.60 (0.26–1.36)	0.217
CRLM characteristics					
	Synchronous CRLM	2.42 (1.18–4.97)	**0.016**	1.72 (0.75–3.92)	0.200
	Tumour Burden Score	1.05 (0.97–1.13)	0.255		
	Tumour number	1.04 (0.95–1.14)	0.404		
	Size of largest tumour size (cm)	1.05 (0.95–1.17)	0.325		
*KRAS* status					
	Impractical *KRAS* (not tested)	1		1	
	*KRAS* Wild-Type	3.15 (0.88–11.32)	0.079	3.12 (0.87–11.19)	0.081
	*KRAS* Mutated	4.27 (1.28–14.17)	**0.018**	4.55 (1.37–15.10)	**0.013**
Surgery procedure					
	Atypical/Anatomical	1.63(0.87–3.08)	0.131		
R1 margin resection status (yes/no)		1.25 (0.63–2.46)	0.521		
Chemotherapy (yes/no)					
	Preoperatively	1.77 (0.95–3.31)	0.073		
	Postoperatively	0.45 (0.24–0.85)	**0.014**	0.42 (0.224–0.801)	**0.008**

CRLM, colorectal liver metastases; HR, Hazard ratio; CI, confidence interval. Bold values are statistically significant (*p* value < 0.05).

## Data Availability

Not applicable.
